# Molecular insights into nagashima-type palmoplantar keratoderma: SERPINB7 mutation spectrum and mechanistic perspectives

**DOI:** 10.3389/fmolb.2026.1796488

**Published:** 2026-06-11

**Authors:** Zhenzhen Xiao, Yue Kang, Rui Li, Yingjian Tan

**Affiliations:** 1 Department of Dermatology, Fuzhou First General Hospital, Fuzhou, China; 2 Department of Respiratory and Critical Care, Xinxiang Central Hospital, Xinxiang, Henan, China; 3 Department of Dermatology, Venereology and Allergology, Charité-Universitätsmedizin Berlin, Berlin, Germany

**Keywords:** founder mutation, gentamicin, LGMN, NPPK, SERPINB7

## Abstract

Nagashima-type palmoplantar keratoderma (NPPK) is a common inherited palmoplantar keratoderma predominantly affecting East Asian populations and caused by biallelic loss-of-function variants in the *SERPINB7* gene. Clinically, NPPK is characterized by diffuse, non-progressive hyperkeratoderma of the palms and soles with erythema extending beyond the palmoplantar margins, frequently accompanied by hyperhidrosis and malodor. Although the disease course is generally benign, these features may lead to a considerable psychosocial burden. Advances in next-generation sequencing have expanded the mutational spectrum of *SERPINB7* and clarified founder effects in different populations, while mechanistic studies have highlighted the essential role of SERPINB7 in maintaining epidermal protease–antiprotease balance and barrier homeostasis. These insights have enabled the development of mutation-targeted therapeutic approaches, most notably topical gentamicin-induced readthrough therapy for nonsense mutations. This review summarizes current knowledge and recent advances in the epidemiology, genetics, pathogenesis, clinical features, and management of NPPK, with particular emphasis on emerging precision treatment strategies.

## 1 Introduction

Inherited palmoplantar keratodermas (PPKs) constitute a heterogeneous group of disorders characterized by abnormal thickening of the skin on the palms and soles (Kabashima et al., 2008; Xiao et al., 2025; Guerra et al., 2018a). Advances in molecular genetics over the last 2 decades have transformed the classification of PPKs from a purely phenotype-based system to one increasingly grounded in genotype and pathomechanism. Within this evolving framework, NPPK has emerged as a paradigmatic example of a common yet previously underrecognized hereditary keratoderma, particularly in East Asian populations (Liu et al., 2023).

NPPK was first described in 1977 by Nagashima as a mild, non-epidermolytic palmoplantar keratoderma with erythema extending beyond the palmoplantar borders. For several decades, the disorder remained poorly defined and was often confused with other diffuse PPKs, most notably mal de Meleda (Kubo, 2025). Due to some overlapping features with Mal de Meleda, such as diffuse transgressive palmar and plantar hyperkeratosis accompanied by palmar and plantar hyperhidrosis, NPPK was initially considered a mild form of Mal de Meleda. It was not until Kabashima and colleagues first reported NPPK and excluded *SLURP1* gene mutations in the patients that NPPK was recognized as an independent type of palmoplantar keratoderma, distinct from Mal de Meleda (Kabashima et al., 2008). This recognition has since facilitated greater awareness and reporting of the disease worldwide. This ambiguity persisted until detailed clinical characterization and genetic studies demonstrated that NPPK represents a distinct nosological entity. The identification of biallelic mutations in *SERPINB7* in 2013 marked a pivotal milestone, firmly establishing NPPK as a genetically defined disease and opening new avenues for mechanistic and therapeutic research (Kubo et al., 2013).

Clinically, NPPK is distinguished by early-onset, diffuse hyperkeratoderma of the palms and soles with relatively mild severity and a non-progressive course (Lyu et al., 2023). Unlike more severe forms of recessive PPK, NPPK does not typically lead to constricting bands, autoamputation, or significant functional impairment. Nevertheless, the frequent presence of hyperhidrosis, secondary infections, and malodor can significantly affect quality of life. As awareness of NPPK has increased, it has become evident that the condition is among the most prevalent inherited PPKs in East Asia, with carrier frequencies suggesting a substantial number of undiagnosed or misdiagnosed individuals (Kazerooni et al., 2025).

From a biological perspective, NPPK has provided important insights into the role of intracellular serine protease inhibitors in epidermal barrier integrity (Huang et al., 2021; Janciauskiene et al., 2024). SERPINB7 belongs to the clade B serpin family, which is thought to protect epithelial cells from inappropriate protease activity induced by endogenous or environmental stressors (Maas and de Maat, 2021; Ohtomo et al., 2008; Kryvalap and Czyzyk, 2022). Loss of SERPINB7 function disrupts this delicate protease–antiprotease balance, leading to impaired barrier function and characteristic clinical manifestations (Lyu et al., 2023). These mechanistic insights not only deepen our understanding of epidermal homeostasis but also highlight potential targets for therapeutic intervention.

In recent years, NPPK has attracted growing attention as a model disease for precision dermatology. The predominance of nonsense mutations in certain populations has prompted clinical trials of topical aminoglycosides aimed at inducing translational readthrough and restoring functional protein expression (Wang et al., 2023; Ohguchi et al., 2018). Although still in an early stage, such approaches exemplify how molecular diagnosis can directly inform targeted treatment strategies in inherited skin diseases.

In this review, we provide a comprehensive and updated overview of NPPK, integrating historical context with recent advances in epidemiology, genetics, molecular pathogenesis, and therapy. By analyzing current evidence and highlighting unresolved questions, we aim to facilitate accurate diagnosis, optimize patient management, and stimulate further research into this common yet often overlooked genodermatosis.

## 2 Clinical phenotype, epidemiology and population genetics

The characteristic feature of NPPK is hyperkeratosis extending beyond the palmar and plantar margins, presenting as well-demarcated, diffuse hyperkeratosis with erythema that can spread to the palms, dorsum of the feet, and Achilles tendon region (Kubo, 2025). The elbows and knees are frequently involved, and hyperhidrosis of the palms and soles is common, predisposing patients to tinea pedis and malodor. Upon water exposure, affected areas develop a white, spongy appearance within 10 min (Kubo et al., 2013). Measurements of transepidermal water loss (TEWL) indicate that lesional skin in NPPK exhibits significantly higher water permeability compared with non-lesional skin or corresponding areas in healthy controls, suggesting that NPPK lesions specifically promote water infiltration into the stratum corneum (Liu et al., 2023). Additionally, NPPK has been reported to co-occur with X-linked ichthyosis, atopic dermatitis, and melanoma, highlighting its potential association with other dermatological conditions (Katayama et al., 2019; Korekawa et al., 2018; Hua et al., 2025; Matsudate et al., 2018).

NPPK exhibits a strikingly uneven global distribution, with the vast majority of reported cases originating from East Asia. Epidemiological studies and large-scale clinical observations indicate that NPPK is one of the most common inherited palmoplantar keratodermas in Japan, China, and Korea (Kubo et al., 2013; On et al., 2017; Brandt et al., 2024). In Japanese populations, the estimated prevalence is approximately 1.2 per 10,000 individuals, while surveys in China suggest an even higher prevalence of around 3.0–3.5 per 10,000 (Liu et al., 2023; Kubo et al., 2013). In contrast, NPPK is exceedingly rare in non-Asian populations, where prevalence estimates are on the order of less than one per million. These marked differences underscore the importance of population-specific genetic factors and founder effects in shaping the global epidemiology of the disease.

No significant sex predilection has been observed in NPPK, and males and females appear to be affected with similar frequency and comparable disease severity (Kubo, 2025). The condition typically manifests in early childhood, often within the first few years of life, although mild cases may go unrecognized until later childhood or adolescence. Seasonal variation in disease severity has not been consistently documented, supporting the notion that NPPK follows a relatively stable, non-progressive clinical course over time (Xiao et al., 2025). Nevertheless, environmental factors such as heat, humidity, and mechanical stress may exacerbate symptoms including hyperhidrosis and malodor, contributing to fluctuations in patient-reported disease burden.

The high prevalence of NPPK in East Asia is largely attributable to founder mutations in the *SERPINB7* gene. The nonsense variant c.796C>T (p.Arg266*) represents the most common pathogenic allele in Japanese and Chinese patients and is widely regarded as an Asian founder mutation (Kubo et al., 2013). Population genetic analyses have demonstrated a relatively high carrier frequency of this allele, which in turn explains the unexpectedly large number of affected individuals despite the autosomal recessive mode of inheritance. In certain regions, this high carrier rate may give rise to an apparent pseudodominant inheritance pattern within families, further complicating clinical recognition and genetic counseling (Kubo, 2025; Kazerooni et al., 2025).

Beyond East Asia, expanding use of next-generation sequencing has led to the identification of NPPK in non-Asian populations, challenging the earlier perception that the disease is almost exclusively confined to East Asians. Notably, several Finnish patients with genetically confirmed NPPK have been reported, all harboring a distinct homozygous missense variant in *SERPINB7* that differs from the Asian founder mutation (Hannula-Jouppi et al., 2020). This observation provides compelling evidence for an independent founder effect in Northern Europe and highlights the role of population bottlenecks and genetic drift in the emergence of rare recessive disorders. To date, sporadic cases have also been documented in other ethnic backgrounds, although the true prevalence outside Asia remains uncertain and is likely underestimated due to limited awareness and diagnostic testing (Brandt et al., 2024).

From a population genetics perspective, NPPK exemplifies how a combination of mild clinical phenotype and high carrier frequency can result in a common yet underdiagnosed genodermatosis. Many affected individuals experience relatively subtle symptoms and may never seek medical attention, while others are misclassified as having nonspecific hyperkeratoderma or fungal infection (Liu et al., 2023). As genetic testing becomes more accessible, particularly through targeted panels for inherited skin diseases and whole-exome sequencing, it is anticipated that additional cases will be identified across diverse populations (Caballero, 2021).

Understanding the epidemiology and population genetics of NPPK has important clinical implications. Accurate prevalence estimates inform healthcare planning and resource allocation, while recognition of founder mutations enables cost-effective genetic screening strategies in high-risk populations (Hannula-Jouppi et al., 2020; Yin et al., 2014). Moreover, population-specific mutation spectra are directly relevant to emerging precision therapies, such as nonsense mutation readthrough approaches, which may be applicable to a substantial proportion of patients in certain ethnic groups (Liu J. et al., 2022). Together, these considerations highlight the need for continued epidemiological surveillance and integration of genetic data into routine dermatological practice.

## 3 Molecular genetics of SERPINB7

### 3.1 Gene identification and disease specificity

NPPK is a genetically well-defined autosomal recessive disorder caused by biallelic pathogenic variants in *SERPINB7*. In 2013, Kubo et al. conducted whole-exome and/or Sanger sequencing in 13 unrelated individuals with NPPK and identified biallelic putative loss-of-function mutations in *SERPINB7*, a cytoplasmic member of the serine protease inhibitor superfamily (Kubo et al., 2013). Among these, c.796C>T (p.Arg266*) was recognized as a major causative founder mutation in both Japanese and Chinese populations. All identified mutations (c.796C>T, c.218_219del2ins12, c.455-1G>A) are predicted to generate premature termination codons upstream of the reactive site, which is essential for protease inhibition, indicating a complete loss of SERPINB7’s protease inhibitory activity in NPPK-affected skin. Since its initial identification as the disease-causing gene in 2013 through whole-exome sequencing studies, *SERPINB7* has been consistently validated as the causative gene for NPPK across diverse ethnic backgrounds (Kubo, 2025).

The *SERPINB7* gene is located on chromosome 18q21.3 and consists of eight exons (Xiao et al., 2025). Alternative splicing gives rise to multiple transcript variants, all of which encode intracellular proteins lacking a signal peptide (Kryvalap and Czyzyk, 2022; Huntington, 2011; Yan et al., 2023). The canonical transcript encodes a 380–amino acid protein that contains the characteristic serpin fold, including three β-sheets, several α-helices, and a C-terminal reactive site loop (RSL) (Miyata et al., 2002; Kelly-Robinson et al., 2021). The RSL is essential for protease inhibition, acting as a pseudosubstrate that forms a covalent complex with target proteases.

As a clade B serpin, SERPINB7 functions primarily within the cytoplasm rather than the extracellular space. This intracellular localization is consistent with its proposed role in protecting keratinocytes from endogenous protease-mediated damage during terminal differentiation and in response to environmental stress (Cohen et al., 2019).

### 3.2 Mutational spectrum of *SERPINB7*

**FIGURE 1:**
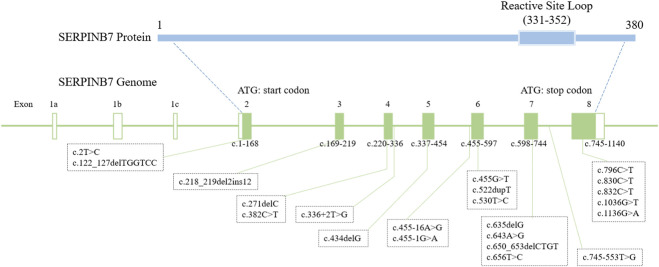
Schematic of SERPINB7 gene and protein with reported mutations in Nagashima-type Palmoplantar Keratoderma. Eight exons (1a–8) and the protein, including the reactive site loop (aa 331–352), are shown. Pathogenic mutations, indicated in cDNA nomenclature (c.), are mapped to their corresponding exons, illustrating the mutation spectrum and affected functional regions.

Since the original discovery of *SERPINB7* mutations in patients with NPPK, more than 22 distinct pathogenic variants have been reported (Table 1; Figure 1) (Liu et al., 2023; Kubo et al., 2013; Xiao et al., 2022; Zhang et al., 2016; Songsantiphap et al., 2020; Hua et al., 2018; Nakajima et al., 2016; Shiohama et al., 2016; Mizuno et al., 2014; Katsuno et al., 2017). These include nonsense mutations, frameshift insertions, deletions, splice-site variants, and missense mutations affecting conserved residues within the serpin domain. Some patients harbor compound heterozygous mutations in *SERPINB7*, whereas others carry homozygous mutations; additionally, digenic mutations involving *SERPINB7* and *SERPINA12* have also been reported (Liu et al., 2023; Kazerooni et al., 2025; Liu et al., 2024). The majority of reported variants are predicted to result in loss of protein function, either through premature truncation, nonsense-mediated mRNA decay, or structural destabilization of the serpin fold.

**TABLE 1:** Reported SERPINB7 mutations in Nagashima-type Palmoplantar Keratoderma.

Mutation of SERPINB7	Genomic location	Predicted protein effect	Mutation type	Reported
c.2T>C	Exon 2	—	Frameshift	Liu et al. (2023)
c.122_127delTGGTCC	Exon 2	p. Leu41_Val42del	Frameshift	Zhang et al. (2016)
c.218_219del2ins12	Exon 3	p. Gln73Leufs* 17	Frameshift	Kubo et al. (2013)
c.271delC	Exon 4	p. His91Thrfs* 9	Frameshift	Hua et al. (2018)
c.336 + 2T>G	Intron 4	—	Splice-site	Mizuno et al. (2014)
c.382C>T	Exon 4	p. Arg128Ter	Nonsense	Katsuno et al. (2017)
c.434delG	Exon 5	p.V146Lfs*12	Nonsense	Liu et al. (2023)
c.455–16A>G	Intron 5	—	Splice-site	Liu et al. (2023)
c.455-1G>A	Intron 5	p. Gly152Valfs* 21	Splice-site	Kubo et al. (2013)
c.455G>T	Exon 6	p. Gly152Val	Nonsense	Yin et al. (2014)
c.522dupT	Exon 6	p.Val175Cysfs*46	Frameshift	Yin et al. (2014)
c.530T>C	Exon 6	p.Phe177Ser	Missense	Xiao et al. (2022)
c.635delG	Exon 7	p. Lys213Serfs* 12	Frameshift	Nakajima et al. (2016)
c.643A>G	Exon 7	p.Asn215Asp	Missense	Xiao et al. (2022)
c.650_653delCTGT	Exon 7	p. Ser217Leufs* 7	Frameshift	Yin et al. (2014)
c.656T>C	Exon 7	p.I219T	Missense	Liu et al. (2023)
c.745–553T>G	Intron 7	—	Splice-site	Liu et al. (2023)
c.796C>T	Exon 8	p. Arg266*	Nonsense	Kubo et al. (2013)
c.830C>T	Exon 8	p. Pro277Leu	Nonsense	Shiohama et al. (2016)
c.832C>T	Exon 8	p.Q278*	Nonsense	Liu et al. (2023)
c.1036G>T	Exon 8	p.E346*	Nonsense	Liu et al. (2023)
c.1136G>A	Exon 8	p. Cys379Tyr	Missense	Hannula-Jouppi et al. (2020)

Among these, the c.796C>T (p.Arg266*) nonsense mutation is by far the most prevalent and represents a well-established founder mutation in East Asian populations, particularly in Japan and China (Kubo, 2025). Additional recurrent variants, such as small deletions or insertions leading to frameshifts, have also been identified and may represent regional or familial mutation hotspots. In non-Asian populations, distinct pathogenic variants have been described, including population-specific founder mutations, supporting genetic heterogeneity on a global scale (Hannula-Jouppi et al., 2020).

Currently, reports on NPPK are mainly concentrated in China and Japan. In 2014, Yin et al. first reported seven cases in China, confirming the founder effect of the c.796C>T mutation in the Han Chinese population (Yin et al., 2014). Subsequently, multiple related cases have been reported domestically. In addition, three cases of NPPK have been reported in Korea (On et al., 2017) and three cases in Finland (Hannula-Jouppi et al., 2020). Analysis of the frequency of the c.796C>T mutation in the 1000 Genomes Project indicates that the heterozygous carrier rate in the general Chinese population is as high as 3%. Therefore, it is inferred that the prevalence of NPPK in the Han Chinese population is approximately 3.1 per 10,000, with more than 300,000 affected individuals. In 2022, Liu et al. conducted the largest cohort study of NPPK to date, including 234 patients (Liu et al., 2023). A total of 14 pathogenic mutations in *SERPINB7* were identified from the cohort. The four most recurrent mutations were c.796C>T (355 alleles, 75.9%), c.522dupT (66, 14.1%), c.650_653delCTGT (24, 5.1%), and c.455G>T (12, 2.6%), accounting for 97.6% of Chinese NPPK patients. Other mutations (11, 2.4%) included c.455-1G>T, c.336 + 2T>G, c.635delG, and seven novel mutations: c.2T>C, c.434delG, c.455-16A>G, c.656T>C, c.745-553T>G, c.832C>T, c.1036G>T. Haplotype analysis revealed that c.522dupT, c.650_653delCTGT, and c.455G>T represent newly identified founder mutations (Liu J. et al., 2022). Another study from China describes eight NPPK patients from seven Chinese Han families, in whom two novel mutations (c.530T>C and c.643A>G) and two recurrent mutations (c.796C>T and c.455G>T) in *SERPINB7* were identified (Xiao et al., 2022). Li et al. reported an 18-year-old male presenting with Nagashima-type palmoplantar keratoderma accompanied by diffuse cerebral white matter abnormalities (Li et al., 2021). Trio-based exome sequencing identified a suspected mosaic compound heterozygous mutation in *SERPINB7*, consisting of a maternally inherited nonsense variant c.796C>T (p.Arg266*) and a *de novo* deletion of exons 4–6. Subsequent copy number variant analysis and low-coverage whole-genome sequencing revealed mosaic deletions spanning 18q21.33–q23. These combined molecular findings explain both the cutaneous and neurological phenotypes and highlight the diagnostic value of integrating multiple genomic approaches in complex genetic disorders.

### 3.3 Genotype–phenotype relationships

Despite the diversity of *SERPINB7* mutations, genotype–phenotype correlations in NPPK appear limited. Patients harboring truncating mutations generally present with a similar clinical phenotype characterized by diffuse, non-progressive palmoplantar hyperkeratoderma with erythema extending beyond the palmoplantar margins (Kubo, 2025; Yin et al., 2014). Missense mutations, although less common, also lead to a comparable phenotype, suggesting that partial loss of SERPINB7 function is sufficient to cross the pathogenic threshold.

Although genotype–phenotype correlations appear limited, this may be explained by a “threshold effect” in SERPINB7 function, whereby different loss-of-function mutations similarly reduce protease inhibitory activity below a critical level required for epidermal homeostasis. As a result, diverse mutations can converge on a relatively uniform clinical phenotype. This concept also implies that therapeutic strategies aimed at partially restoring SERPINB7 activity or rebalancing downstream protease signaling may be sufficient to achieve clinical benefit, regardless of the specific mutation type.

This relative phenotypic uniformity implies that NPPK is a threshold disorder, in which disease manifests once SERPINB7 activity falls below a critical level, rather than through mutation-specific dominant-negative effects (Kazerooni et al., 2025). This concept has important implications for therapeutic development, as even modest restoration of SERPINB7 expression or activity may confer clinical benefit.

### 3.4 *SERPINA12*-related PPK similar to NPPK

In 2020, loss-of-function variants in *SERPINA12*, another SERPIN expressed in the epidermis, were reported to cause autosomal recessive PPK, which phenotypically resembles NPPK(Mohamad et al., 2020). Notably, in both NPPK and *SERPINA12*-related PPK, patient tissues did not show abnormal aggregates or polymers of pathogenic SERPIN proteins (Brandt et al., 2024; Liu Y. et al., 2022; Li Z. et al., 2024). This suggests that the disease phenotype arises not from accumulation of mutant SERPINs, but from overactivation of their target proteases due to the reduced inhibitory activity of SERPINB7 and SERPINA12. Given the phenotypic similarity between NPPK and *SERPINA12*-associated PPK, it is reasonable to hypothesize that these two protease inhibitors share overlapping localization and functions relevant to pathogenesis (Kubo, 2025). Multiple research groups have reported that compound heterozygous mutations in SERPINB7 and SERPINA12 can cause NPPK, further suggesting that these two protease inhibitors may contribute to PPK through similar pathogenic mechanisms (Liu et al., 2024; Jiang et al., 2024; Huang et al., 2025).

Reduced SERPINA12 expression has been shown to diminish inhibition of kallikrein 7, as well as to decrease levels of desmoglein-1 and corneodesmosin, both established substrates of kallikrein 7 (Mohamad et al., 2020). These findings indicate that SERPINA12 is secreted by keratinocytes and functions to protect the corneodesmosome from proteolytic degradation by restraining kallikrein activity in the extracellular space of the stratum corneum. Consequently, SERPINA12 deficiency likely contributes to stratum corneum barrier defects through excessive degradation mediated by uncontrolled kallikrein proteases.

*SERPINA12*-associated PPK and *SERPINB7*-related NPPK are clinically difficult to distinguish, and both follow an autosomal recessive inheritance pattern. Whether these two conditions represent the same disease entity remains to be further elucidated (Liu et al., 2024). In general, the inflammatory phenotype associated with SERPINA12 deficiency–related PPK appears to be slightly more pronounced, which may be related to the proposed anti-inflammatory function of SERPINA12 (Zhang et al., 2025). In addition, several cases of NPPK potentially resulting from digenic inheritance involving *SERPINA12* and *SERPINB7* have been reported, suggesting that these two proteins may contribute to disease pathogenesis through shared mechanisms, possibly involving common substrate proteases (Liu et al., 2024; Huang et al., 2025). These issues require further clinical and experimental studies to be fully clarified.

## 4 Genetic and molecular mechanisms of NPPK

The pathogenic mechanism of SERPINB7 mutations in NPPK has long remained elusive. Previous studies primarily hypothesized that SERPINB7 deficiency might disrupt the balance of protease activity in the epidermis, leading to impaired skin barrier function. Such disruption could facilitate the invasion of bacteria, fungi, and viruses, while also promoting hyperkeratosis and epidermal thickening through abnormal keratinocyte differentiation (Liu et al., 2023).

The proper function of a protein relies on the integrity of its core functional domains. Most of the pathogenic mutations identified in *SERPINB7* introduce premature stop codons, leading to truncated proteins that lack the RSL region, thereby abolishing their functional activity (Morizane et al., 2022). The study also demonstrated that both nonsense-mediated mRNA decay (NMD) and 26S proteasome–mediated protein degradation contribute jointly to the reduction of intracellular levels of the truncated protein resulting from the SERPINB7 c.796C>T mutation. Current evidence does not clearly distinguish between aberrant expression patterns and structural disruption, and both mechanisms remain plausible explanations.

Finnish researchers identified a novel missense mutation, c.1136G>A. Immunohistochemical analysis of lesional skin from patients homozygous for this mutation showed that the mutant SERPINB7 protein was not only highly expressed in the granular layer but also aberrantly expressed in the spinous layer (Hannula-Jouppi et al., 2020). Structural modeling predicted that the amino acid substitution caused by this mutation is located near the RSL region, potentially affecting its mobility. The mutation may lead to loss of function through disruption of critical SERPINB7 domains, such as the RSL or CD-loop, reduced RSL mobility, or formation of nonfunctional aggregates, ultimately resulting in the deficiency of normal intracellular SERPINB7 protein (Suzuki et al., 2015).

In 2025, Li et al. provided the first direct evidence identifying Legumain as the protease specifically inhibited by SERPINB7(Li S. et al., 2024). Loss of SERPINB7 function in the skin results in increased Legumain activity, leading to excessive degradation of structural proteins, including intermediate filaments, within keratinocytes. This proteolytic imbalance likely contributes to barrier dysfunction, manifesting clinically as hyperkeratosis and spongiosis in the palmar and plantar regions. Importantly, Legumain emerges as a potential therapeutic target for NPPK. This study significantly advanced our understanding of protease–protease inhibitor networks in the skin and opened new avenues for research into keratinization disorders.

Further elucidating this pathway, the 2026 study by Ma et al. revealed that SERPINB7 mutations not only regulate Legumain activity but also affect O-GalNAc glycosylation (Ma et al., 2025). This work identified a SERPINB7–Legumain–O-GalNAc glycosylation axis as a critical regulator of skin barrier homeostasis. Mechanistically, SERPINB7 modulates O-GalNAc glycosylation in keratinocytes, influencing calcium signaling and cell adhesion, thereby providing a molecular explanation for its role in maintaining epidermal integrity. Moreover, this study linked defects in O-GalNAc glycosylation to inflammatory skin disorders, suggesting that targeting SERPINB7, Legumain, or glycosylation-related enzymes could offer novel therapeutic strategies for conditions such as psoriasis and atopic dermatitis.

A recent study characterized a deep intronic *SERPINB7* founder variant in six Chinese NPPK patients and demonstrated its pathogenicity (Chen et al., 2025). This variant causes the inclusion of a pseudo-exon, resulting in the production of a truncated, dysfunctional SERPINB7 protein. Mechanistically, the abnormal splicing is mediated by excessive binding of the splicing factor SRSF9 to the mutant transcript. Functional studies using plasmid and minigene constructs confirmed the loss of SERPINB7 activity due to aberrant splicing. Importantly, an antisense oligonucleotide (ASO) targeting this variant successfully corrected the splicing defect *in vitro*, highlighting a potential precision therapy approach. Haplotype analysis further confirmed that this deep intronic mutation represents a Chinese founder variant, providing both mechanistic insight and a therapeutic target for NPPK.

Di et al. reported that, following *SLURP1* mutations, mechanical stress led to reduced SERCA2b activity in keratinocytes, accompanied by elevated cytosolic calcium levels and activation of the PERK–NRF2 signaling pathway, ultimately resulting in epidermal thickening in patients with palmoplantar keratoderma (Di et al., 2025). These findings suggest that the clinical phenotype of NPPK may also be influenced by mechanical stress, although direct evidence and the underlying molecular mechanisms remain to be further elucidated.

SERPINB7 is essential for maintaining epidermal homeostasis by regulating keratinocyte differentiation and barrier integrity. Loss of SERPINB7 abolishes its inhibitory effect on the cysteine protease LGMN, leading to excessive LGMN activity that contributes to the pathogenesis of NPPK (Li S. et al., 2024). This dysregulation impairs O-GalNAc glycosylation, weakening keratinocyte cohesion, while simultaneously reducing intracellular calcium influx required for terminal differentiation and the expression of key barrier proteins such as filaggrin and loricrin (Ma et al., 2025). The resulting barrier dysfunction facilitates the penetration of environmental antigens. It amplifies cutaneous immune responses, thereby linking SERPINB7 deficiency not only to NPPK but also to increased susceptibility to inflammatory skin diseases such as psoriasis and atopic dermatitis (Hua et al., 2025; Zheng et al., 2022).

Together, these findings illuminate the central role of SERPINB7 in orchestrating protease activity and post-translational modification within keratinocytes, bridging a long-standing gap in the mechanistic understanding of skin barrier regulation in NPPK.

## 5 Clinical management and therapeutic strategies

At present, the management of NPPK is largely symptomatic, as no curative therapy is available (Gram et al., 2024). Emollients and moisturizers remain the cornerstone of treatment, aiming to improve skin hydration, soften hyperkeratotic lesions, and alleviate discomfort. Topical keratolytic agents, such as urea or salicylic acid, are often used adjunctively to reduce excessive scaling (Guerra et al., 2018b). Topical retinoids may help normalize keratinocyte differentiation and reduce epidermal thickening, although their use is frequently limited by local irritation. Topical corticosteroids are occasionally prescribed to control secondary inflammation or erythema, but their long-term efficacy in NPPK is modest and primarily supportive (Bodemer et al., 2021).

Beyond conventional therapies, increasing attention has been directed toward gentamicin-mediated read-through therapy, which represents a mechanism-based therapeutic approach for NPPK patients harboring nonsense mutations in *SERPINB7*(Pasmooij, 2018). Gentamicin, an aminoglycoside antibiotic, has been shown to induce ribosomal read-through of premature termination codons, thereby partially restoring full-length SERPINB7 protein expression. Several studies have demonstrated that topical gentamicin treatment can significantly improve hyperkeratosis and erythema in NPPK, particularly in patients with nonsense mutations, whereas individuals with missense mutations show little or no response (Ohguchi et al., 2018). Importantly, the therapeutic effect appears to be reversible upon treatment discontinuation, highlighting the need for sustained application. In 2018, a total of 20 patients with genetically confirmed NPPK were enrolled in a 30-day double-blind, randomized controlled trial. Compared with placebo, gentamicin ointment significantly improved hyperkeratosis and malodor, with no statistically significant difference observed between the 0.1% and 0.3% concentrations. However, its effect on erythema was minimal (Wang et al., 2023). These results suggest that gentamicin ointment has potential as a therapeutic option for NPPK, warranting further investigation.

Although gentamicin-mediated readthrough therapy and ASO-based correction strategies have been proposed as promising approaches for *SERPINB7*-related disorders, their limitations and translational challenges should be carefully considered. The efficiency of readthrough therapy varies significantly depending on the specific premature stop codon and its surrounding sequence context, which may lead to heterogeneous therapeutic outcomes among patients with different nonsense mutations (Ohguchi et al., 2018). In addition, the topical use of aminoglycosides raises concerns regarding potential local toxicity and off-target effects, particularly with prolonged application. For ASO-based approaches, effective delivery into the thickened palmoplantar epidermis remains a major obstacle, potentially limiting their clinical efficacy (Chen et al., 2025). Furthermore, long-term safety and durability of these interventions have not yet been fully established. Addressing these challenges will be essential for advancing these strategies toward clinical application and ensuring their safety and effectiveness. Nevertheless, this approach provides a compelling proof of concept for precision medicine in inherited keratinization disorders and underscores the potential of targeting the underlying genetic defect in NPPK.

## 6 Functions of SERPINB7 in psoriasis and atopic dermatitis

**FIGURE 2:**
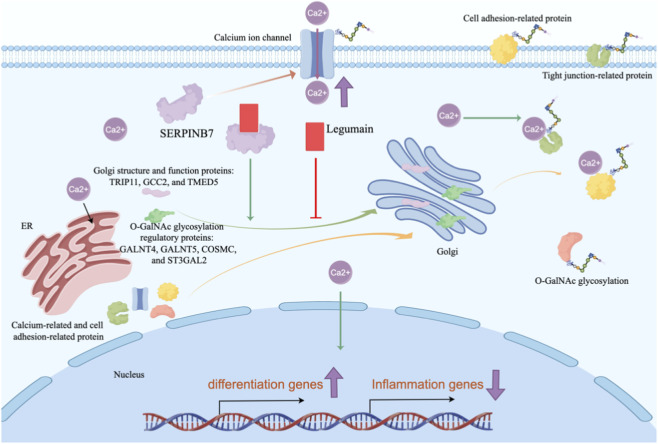
SERPINB7 regulates epidermal homeostasis through LGMN inhibition, glycosylation, and calcium signaling SERPINB7 restrains LGMN protease activity, thereby preserving proper O-GalNAc glycosylation and maintaining keratinocyte cohesion and structural integrity. In parallel, SERPINB7 supports intracellular calcium influx required for keratinocyte terminal differentiation and the expression of key barrier proteins such as filaggrin and loricrin. Through these coordinated pathways, SERPINB7 ensures epidermal barrier stability, limits antigen penetration, and helps prevent excessive cutaneous immune activation.

Clinically, loss-of-function mutations in *SERPINB7* cause NPPK, and a proportion of affected individuals present with concomitant atopic dermatitis, suggesting that SERPINB7 deficiency may predispose to allergic skin inflammation in the context of epidermal barrier dysfunction (Figure 2) (Hua et al., 2025). Mechanistically, SERPINB7 is essential for maintaining keratinocyte differentiation and epidermal integrity. Its deficiency leads to reduced intracellular calcium influx in keratinocytes, resulting in decreased expression of differentiation-associated proteins such as loricrin, filaggrin, and keratin 10, while simultaneously increasing the expression of pro-inflammatory chemokines (CXCL1, CXCL2, and CCL20) and antimicrobial peptides (S100A7, S100A8, and S100A12), thereby promoting a psoriasis-like inflammatory phenotype (Zheng et al., 2022). At the molecular level, SERPINB7 exerts its function in part through inhibition of the cysteine protease legumain, which is required to maintain proper O-GalNAc glycosylation in keratinocytes (Ma et al., 2025). This modification is critical for keratinocyte differentiation and cell–cell adhesion, and its disruption following SERPINB7 loss contributes to epidermal barrier impairment and inflammation. In addition, large-scale genetic evidence supports its involvement in atopic dermatitis: a genome-wide meta-analysis of 796,661 individuals from the FinnGen study, the Estonian Biobank, and the UK Biobank identified *SERPINB7* as a potential susceptibility locus (Sliz et al., 2021). Consistently, transcriptomic analyses have shown that SERPINB7 expression is elevated in lesional compared with non-lesional skin in psoriasis, and the gene is located in proximity to psoriasis-associated loci, further supporting its role in disease pathogenesis (Wang et al., 2018).

## 7 Conclusion and prospective

NPPK is a rare inherited keratinization disorder whose molecular pathogenesis has only recently begun to be elucidated. Accumulating evidence highlights the central role of SERPINB7 in maintaining epidermal homeostasis through the regulation of protease activity, post-translational modifications, and skin barrier integrity. The identification of Legumain as a direct target protease of SERPINB7, together with the discovery of the SERPINB7–Legumain–O-GalNAc glycosylation regulatory axis, has significantly advanced our understanding of the disease mechanism and provided novel insights into epidermal barrier regulation. The identification of founder mutations in *SERPINB7* has important implications for the diagnosis of NPPK, particularly in populations with a high carrier frequency. It enables more efficient and cost-effective genetic screening by prioritizing common recurrent variants. Moreover, recognizing these founder mutations facilitates early diagnosis and genetic counseling, especially in endemic regions (Yin et al., 2014).

Several important questions regarding SERPINB7 remain unresolved and warrant further investigation. First, it is unclear why different loss-of-function mutations in *SERPINB7* lead to a relatively consistent clinical phenotype, such as that observed in NPPK, suggesting convergence on shared downstream mechanisms that remain to be defined. Second, the interaction between mechanical stress and SERPINB7 deficiency is poorly understood, particularly given the predilection of lesions for palmoplantar regions exposed to constant friction and pressure. It remains to be clarified how mechanical stimuli may exacerbate protease dysregulation and barrier impairment in this context. Third, the therapeutic potential of targeting this pathway is still largely unexplored; specifically, whether inhibition of LGMN or modulation of O-GalNAc glycosylation can restore keratinocyte differentiation and barrier function requires further evaluation. Finally, robust *in vivo* models are needed to validate these mechanisms and to establish causal links between SERPINB7 deficiency, protease dysregulation, and epidermal barrier dysfunction in a physiologically relevant setting.

Current therapeutic approaches for NPPK remain largely symptomatic; however, gentamicin-mediated read-through therapy represents an important step toward mechanism-based and mutation-specific treatment. While its clinical application is currently limited to patients with nonsense mutations, this strategy underscores the potential of precision medicine in inherited skin disorders. Looking forward, further investigation into *SERPINB7*-associated signaling pathways, protease networks, and glycosylation processes may facilitate the development of targeted therapies not only for NPPK but also for other inflammatory and hyperkeratotic skin diseases. Continued integration of basic research and clinical studies will be essential to translate these mechanistic insights into durable and effective therapeutic interventions.
