# Correction: Molecular insights into nagashima-type palmoplantar keratoderma: SERPINB7 mutation spectrum and mechanistic perspectives

**DOI:** 10.3389/fmolb.2026.1909877

**Published:** 2026-07-13

**Authors:** Zhenzhen Xiao, Yue Kang, Rui Li, Yingjian Tan

**Affiliations:** 1 Department of Dermatology, Fuzhou First General Hospital, Fuzhou, China; 2 Department of Respiratory and Critical Care, Xinxiang Central Hospital, Xinxiang, Henan, China; 3 Department of Dermatology, Venereology and Allergology, Charité-Universitätsmedizin Berlin, Berlin, Germany

**Keywords:** founder mutation, gentamicin, LGMN, NPPK, SERPINB7

There was a mistake in Figure 2 as published. In Figure 2, SERPINB7 was incorrectly labeled as SERPINA12. The corrected Figure 2 appears below.

**FIGURE 2:**
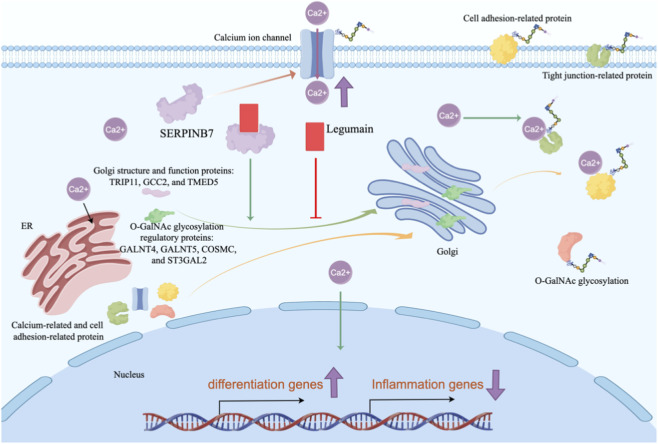
SERPINB7 regulates epidermal homeostasis through LGMN inhibition, glycosylation, and calcium signaling SERPINB7 restrains LGMN protease activity, thereby preserving proper O-GalNAc glycosylation and maintaining keratinocyte cohesion and structural integrity. In parallel, SERPINB7 supports intracellular calcium influx required for keratinocyte terminal differentiation and the expression of key barrier proteins such as filaggrin and loricrin. Through these coordinated pathways, SERPINB7 ensures epidermal barrier stability, limits antigen penetration, and helps prevent excessive cutaneous immune activation.

The original version of this article has been updated.

